# Taping after Rhinoplasty for Asian Noses

**Published:** 2013

**Authors:** Kianoosh Nahid, Chong AW

**Affiliations:** 1 *MS ORL-HNS (UM), Department ofOtorhinolaringology, Faculty of Medicine Building, University ofMalaya, Kuala Lumpur, Malaysia*

**Fig1:**
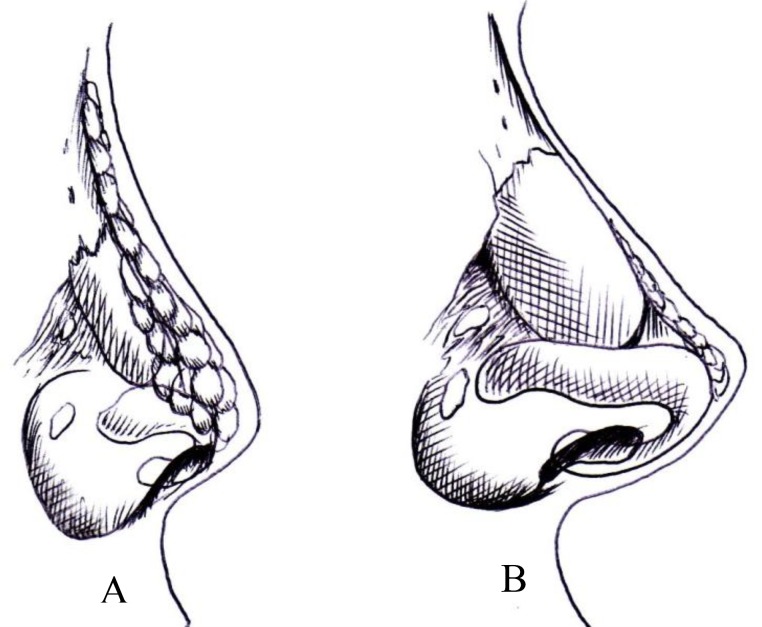
Differences between Asian Nose (A) and Caucasian Nose (B), drawing by Author

Nasal anatomy and shape is race-related. Caucasians (leptorrhine) have generally high dorsum and projected tip, while The Asian nose (platyrrhine, mesorrhine) is known by thick skin with low, wide and flat nasal bridge with short nasal bones, and a broad, bulbous, thick-skinned and less projected nasal tip (Fig 1).

**Fig2:**
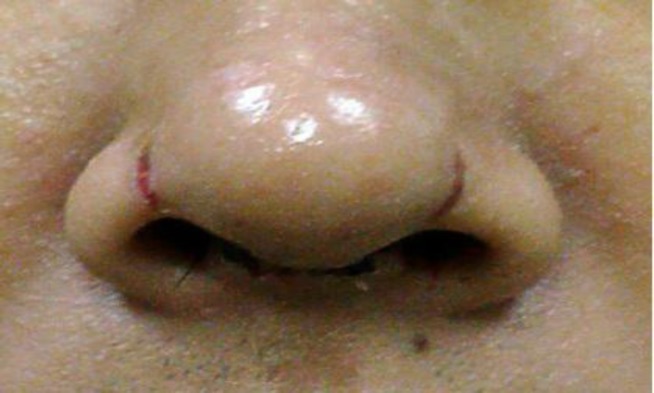
One week post op view: pressure-sore wound on nasal soft triangles

Taping is considered the main support for newly modified structures of nose after rhinoplasty, long after other splints like thermoplastic or plaster casts have been removed a few days post op. Usually the patient is instructed to personally change the tapes after the first few sessions of being visited by the rhinosurgeon. Taping can potentially be harmful if applied and removed improperly. Examples are detaching the skin from nasal framework during pulling off the tape and consequent subcutaneous bleeding, or ischemic injury in nasal soft tissue triangle. We experienced the latter complication in two of our rhinoplasty Asian patients. (Fig 2).

**Fig3:**
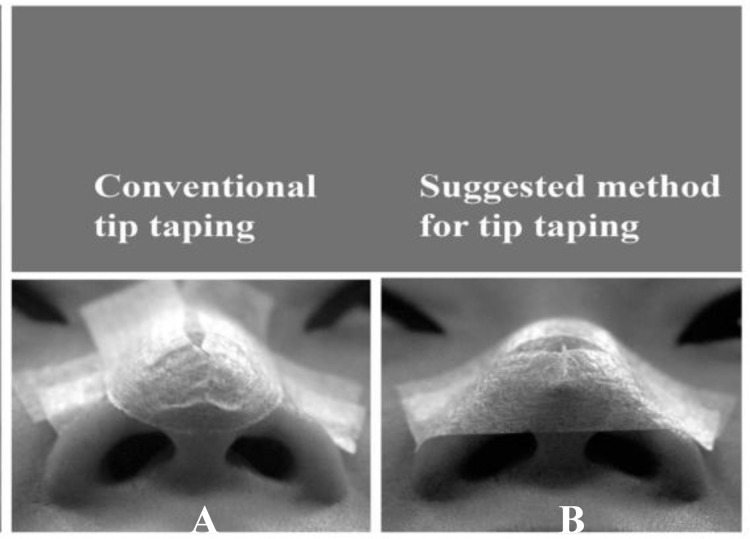
Side view of an Asian nose and (to be removed) conventional taping method as well as the suggested one

Realizing that the taping method (sling around the nasal tip as being done for Caucasian noses) was the cause of ischemic trauma, some changes were applied to the original way of U shape sling under the nasal tip in further tapings for these patients. The aim was to change the shape of sling from U to V to make sure the edges of tape wouldn’t be located in the nasal soft triangle (Fig 3). The injuries were healed in a month.

We believe that during the U shape taping for Asian noses, the lateral edge of tape can cause pressure-sore on soft triangle. Hence the taping method is better to be modified from U shape around the tip to V not to “narrow” the nasal tip and just support the lower part of it. We have followed this modified way of taping for our following Asian patients and haven’t encountered any similar problem ever since. 

So, for taping of Asian noses, we suggest: “Do the taping in a fashion that half of the tape (especially the first layer) remains on the nasal tip and the other half on the alae to avoid unwanted trauma to soft triangle.” 

